# Effects of cerebral perfusion pressure on regional cerebral blood flow in dogs with acute epidural hematoma: quantitative evaluation with contrast-enhanced ultrasound

**DOI:** 10.18632/oncotarget.21795

**Published:** 2017-10-11

**Authors:** Hongwei Cheng, Xiang Mao, Zonggang Hou, Jian Xu, Shuyu Hao, Huan Li, Baiyun Liu

**Affiliations:** ^1^ Department of Neurosurgery, The First Affiliated Hospital of Anhui Medical University, Hefei, Anhui, China; ^2^ Neurotrauma Laboratory, Beijing Neurosurgical Institute, Capital Medical University, Beijing, China; ^3^ Department of Neurosurgery, Beijing Tian Tan Hospital, Capital Medical University, Beijing, China; ^4^ Nerve Injury and Repair Center of Beijing Institute for Brain Disorders, Beijing, China; ^5^ China National Clinical Research Center for Neurological Diseases, Beijing, China

**Keywords:** cerebral perfusion pressure, regional cerebral blood flow, intracranial pressure, contrast-enhanced ultrasound, Pathology Section

## Abstract

To discuss the relationship between the regional cerebral blood flow (rCBF) and cerebral perfusion pressure (CPP) and the effect of CPP on rCBF in different spaces in an experimental animal model. As the ICP increased, the CPP and rCBF (A × β value measured by CEU) decreased to varying degrees. The rCBF_1_ and rCBF_2_ were well correlated with the CPP. At the same CPP, rCBF_1_ decreased significantly than the level of rCBF_2_ (p < 0.01). Six healthy cross-breed dogs, both males and females, weighing 18.3 ± 1.6 kg, were selected to establish models of increased intracranial pressure (ICP) via the installation of an epidural latex sacculus. The calculated CPP was in accordance with the ICP through the formula CPP = MAP - ICP, and contrast-enhanced ultrasound (CEU) was used to instantly measure the rCBF 1 and 2 cm around the sacculus edge. The relationship between rCBF 1 cm (rCBF_1_) and 2 cm (rCBF_2_) around the sacculus edge and the CPP was analyzed. As the ICP increased, the CPP and rCBF both decreased. The rCBF and the CPP had a linear relationship, but the perfusion pressure did not necessarily determine all parts of the rCBF. The rCBF was different in different spaces: the farther away from the injured site, the smaller the effect on the rCBF.

## INTRODUCTION

Traumatic brain injury (TBI) has the highest mortality and disability rates among all human traumatic diseases and is responsible for more than half of all traumatic deaths [1]. TBI can be classified into primary and secondary brain injuries. Primary brain injury is irreversible and can develop/deteriorate into secondary brain injury involving a complex pathophysiological process; secondary injuries include cerebral ischemia, brain edema, and cerebral compression caused by space-occupying hematoma, among other conditions. Secondary brain injury can be minimized by clinical intervention, which is the goal of modern neurosurgery in treating TBI. However, no fundamental breakthroughs have been achieved in clinical intervention to treat complex hemodynamic changes caused by a post-traumatic increase in intracranial pressure (ICP).

Post-traumatic cerebral ischemia is still a focus in TBI research. Graham [2] performed autopsies on 151 patients who died of TBI from 1968-1972 and found that 91% of them had brain ischemic damage. Hakin [3] proposed that when the regional cerebral blood flow (rCBF) is below 20 ml/100 g/min, an ischemic penumbra of brain tissue emerges and is characterized by cessation of interneuron signal transmission, reduction in cellular aerobic metabolism, suppression of ion pump function, neuron depolarization, and increased production of anaerobic glycolytic products (e.g., lactic acid); when rCBF is below 10-12 ml/100 g/min, the resulting discontinued ATP synthesis and functional failures of ion pumps could lead to irreversible necrosis of brain tissue. Therefore, rCBF can serve as an important parameter to describe cerebral ischemia. Cerebral perfusion pressure (CPP) plays a pivotal role in maintaining normal cerebral blood flow (CBF) because CBF is mainly governed by the cerebral vascular resistance (CVR) and CPP. Under pathological conditions, the maintenance of CPP and CBF at certain levels is prioritized via the self-regulation of cerebral vessels to meet the metabolic requirements of brain tissue. After a large number of experiments, Rosner [4] proposed that improving CPP should be the major focus in the treatment of traumatic intracranial hypertension and emphasized the importance of maintaining CPP and CBF at certain levels. Muizelaar [5] revealed that increasing blood pressure and CPP could lead to marked CBF improvement but a negligible change in ICP and that ICP often did not increase with increases in the CPP. To date, the mechanisms underlying the complex CBF dynamic changes in the acute phase after TBI remain unknown. Clarifications of the questions regarding the changing pattern of rCBF and the specific relationship between rCBF and CPP with an increase in ICP and decrease in CPP are undoubtedly important for guiding clinical treatments. However, the research on CPP and rCBF in the acute phase of TBI is very limited.

Employing a domestic dog model of epidural hematoma in combination with ventricular ICP and electrocardiogram (ECG) monitoring, the present study used contrast-enhanced ultrasonography (CEU) to detect rCBF changes at different distances from the compression site of cerebral tissue and investigated the relationship between the changes in rCBF and CPP. Our findings provide a foundation for further research and the development of effective clinical interventions.

## RESULTS

### Model

**Figure 1:**
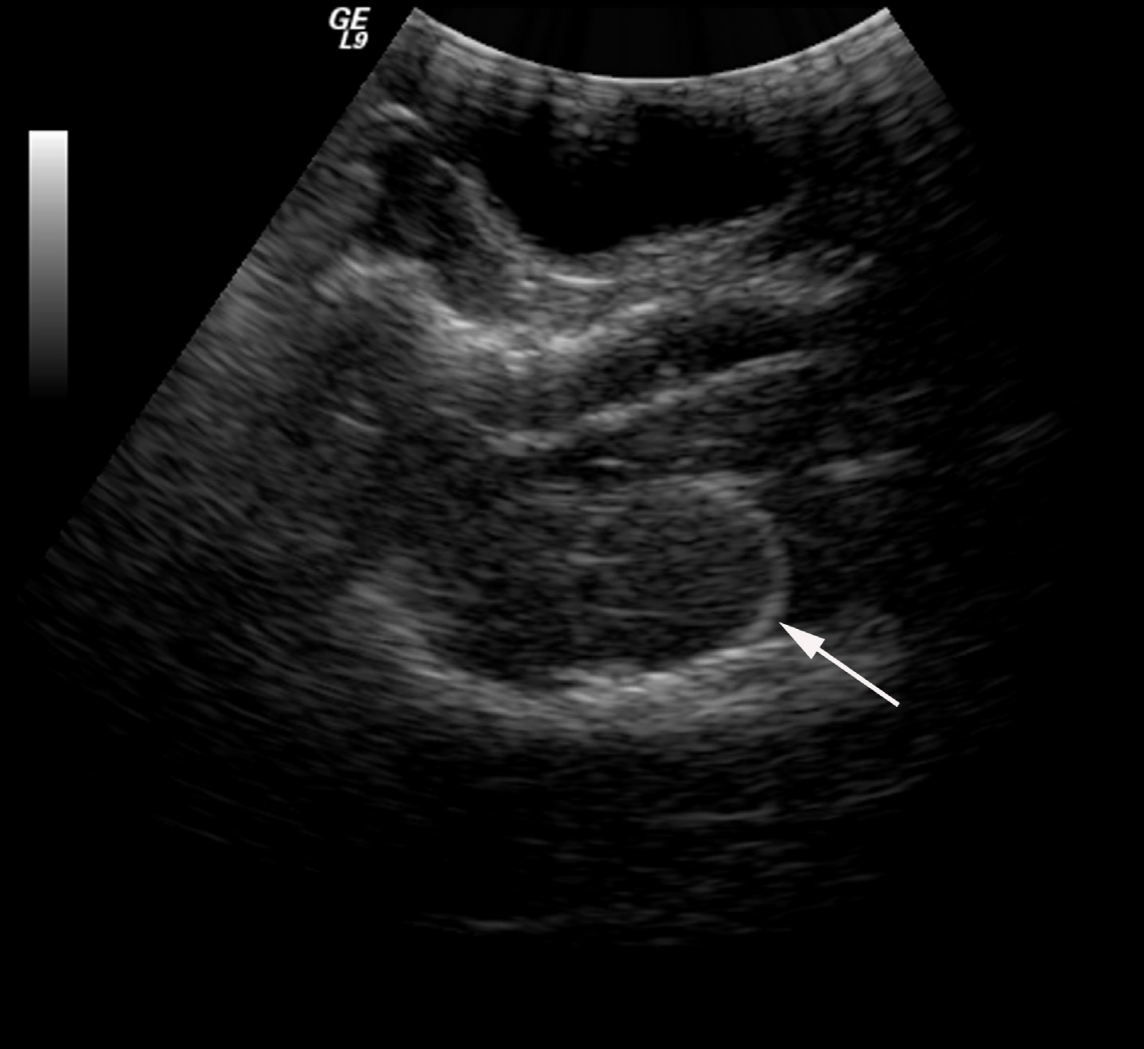
An epidural hematoma model was established, and the position of sacculus was confirmed *via* ultrasound (white arrow)

In this study, an epidural hematoma model was successfully established (Figure 1). The MAP of the experimental dogs was at 88.7 ± 14.3 mmHg before trauma (the volume of sacculus was 0ml) and the MAP was 98.2±8.1 mmHg (the volume of sacculus was around 9ml) before the CPP reached nearly 0 mmHg. A linear relationship between CPP and ICP was found and described by the equation CPP = MAP-ICP. Other vital signs of the experimental dogs were generally stable.

### Relationship between ICP and time

**Figure 2:**
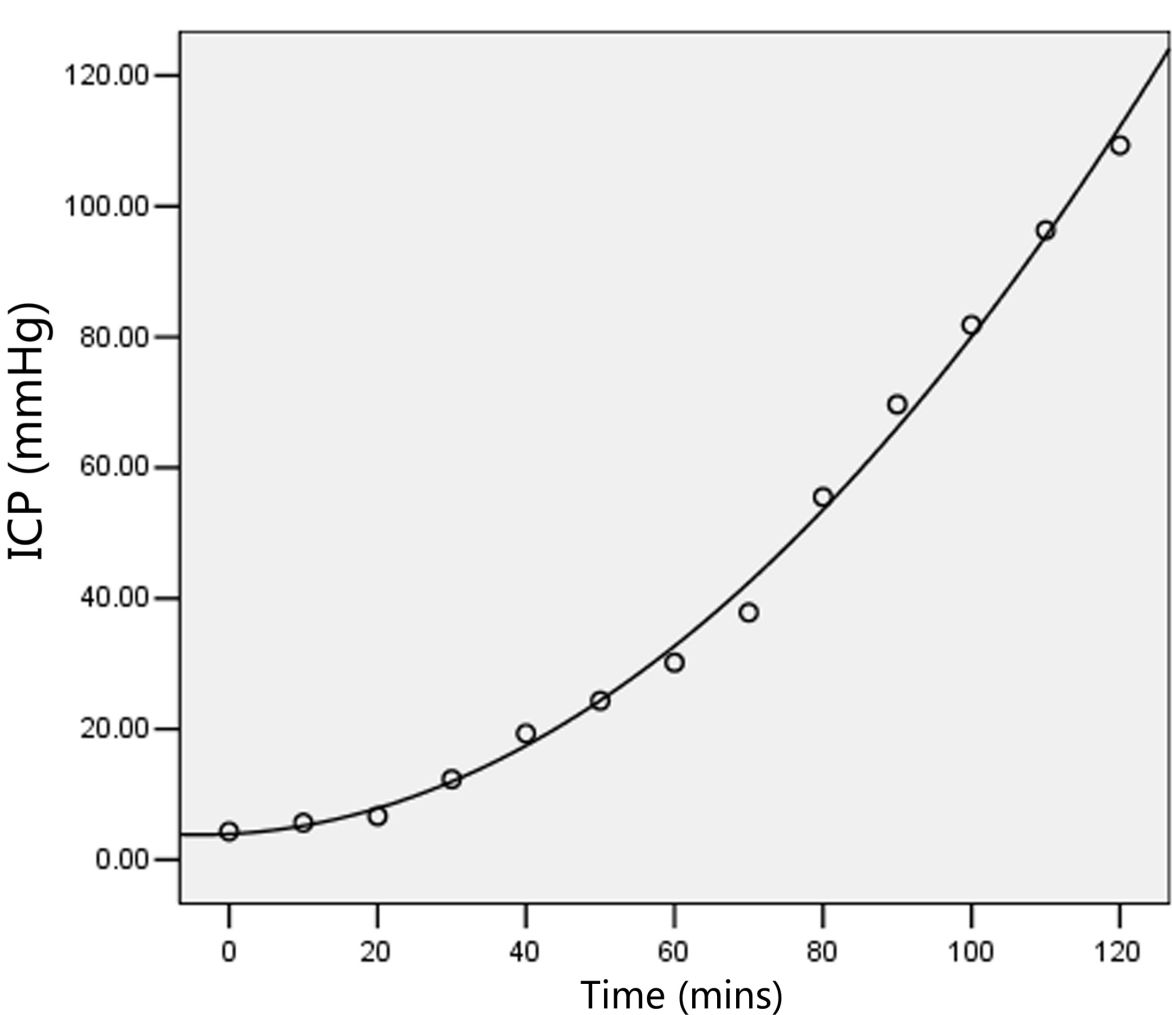
The dependence of ICP on time followed a cubic power law

Using SPSS 19.0, for values of p<0.05, the goodness-of-fit of the correlation was analyzed, and the most accurate regression curve was selected to generate the images (Figure 2) and equations below: ICP = 5.13 - 0.93 × t + 0.10 × t^2^ - 1.80 × t^3^. The results indicated that the dependence of ICP on time followed a cubic power law.

### Relationship between ICP and CBF

**Figure 3:**
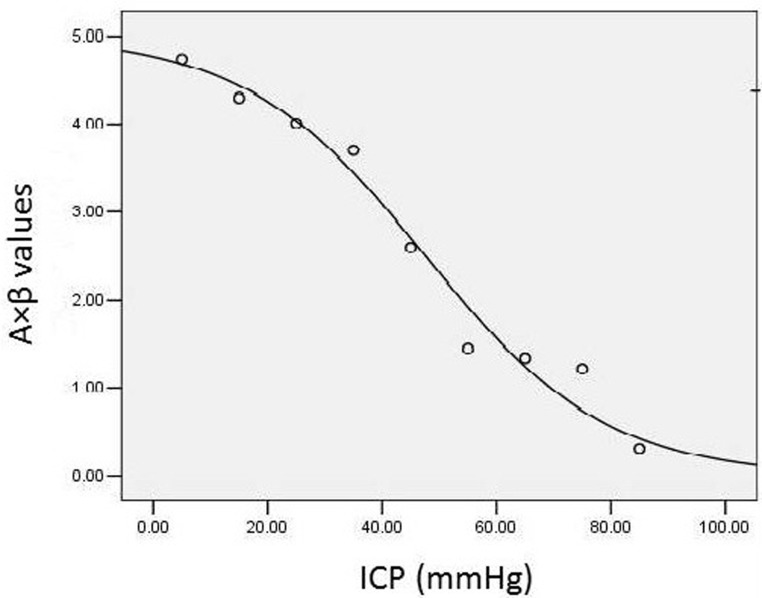
A logistic regression equation was selected based on the goodness-of-fit R^2^ and F values for the relationship between ICP and rCBF

The image analysis software for contrast echocardiography automatically provided CEU parameters, including the A, β, and A×β values, for the exponential curve fitting equation. The correlation analysis yielded the results of r = -0.980 and p < 0.001, indicating a close correlation between the two parameters. Subsequently, a logistic regression equation was selected based on the goodness-of-fit R^2^ and F values (R^2^ = 0.960, F = 198.454, p < 0.001, Fig. 3). The value of A×β, which represents rCBF, decreased to varying extents with increasing ICP and decreasing CPP (Figure 3).

### Relationship between rCBF and CPP

The curvilinear regression analysis of the data, as measured using SPSS 19.0, revealed satisfactory linear correlations of both rCBF_1_ (the rCBF at the site 1 cm away from the compression site) and rCBF_2_ (the rCBF at the site 2 cm away from the compression site) with the CPP. Similarly, both rCBF_1_ and rCBF_2_ showed satisfactory linear correlations with the change in CPP.

**Figure 4:**
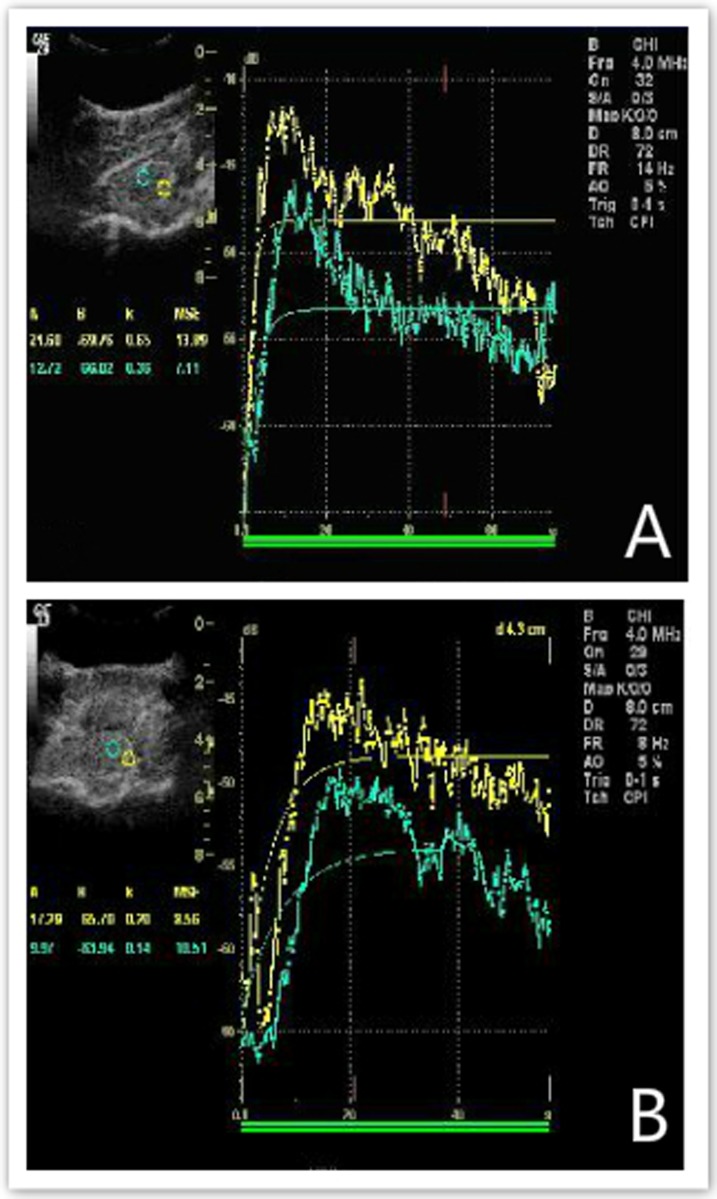
The rCBF values at sites 1 and 2 cm away from the compression site both decreased to varying extents **A.** shows the rCBF values, including the A, β, and A×β values, at the sites 1 and 2 cm away from the compression site under CPP = 84 mmHg; **B.** shows the rCBF values, including A, β, and A×β values, at the sites 1 and 2 cm away from the compression site under CPP = 29 mmHg.

**Figure 5:**
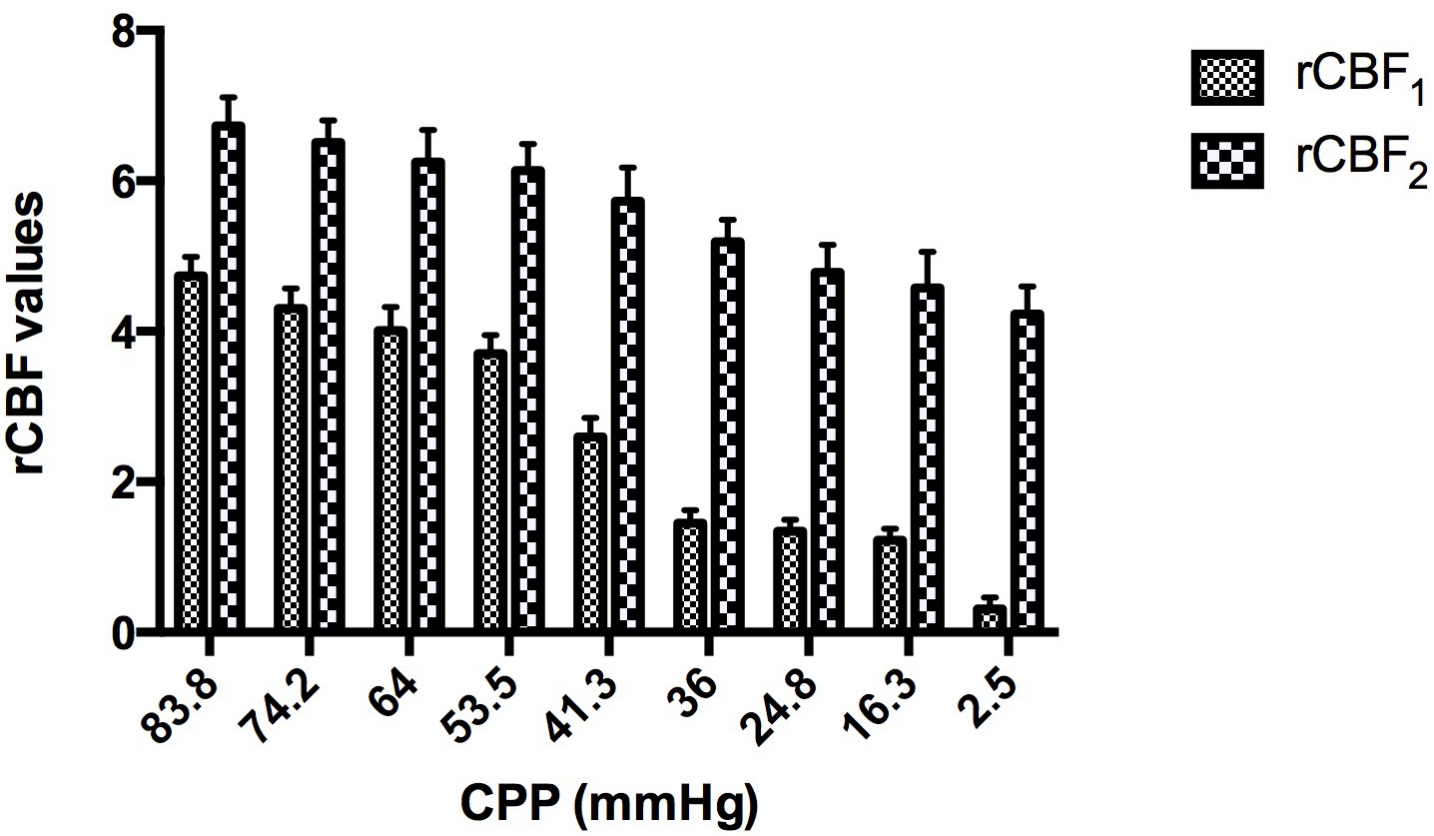
The rCBF values at the sites 1 and 2 cm away from the compression site both decreased to varying extents

With increasing ICP, the CPP decreased accordingly, and the rCBF values at the sites 1 and 2 cm away from the compression site both decreased to varying extents (Figure 4). In addition, the value of rCBF_1_ was markedly lower than rCBF_2_ under the same CPP, and the difference between the two was statistically significant (Figure 5, p < 0.01). The equations were as follows rCBF_1_ = 0.085 + 0.058 * CPP (R2 = 0.953, F = 142.597, p < 0.001) and rCBF_2_ = 4.124 + 0.033 * CPP (R2 = 0.970, F = 224.207, p < 0.001).

### Staining

**Figure 6:**
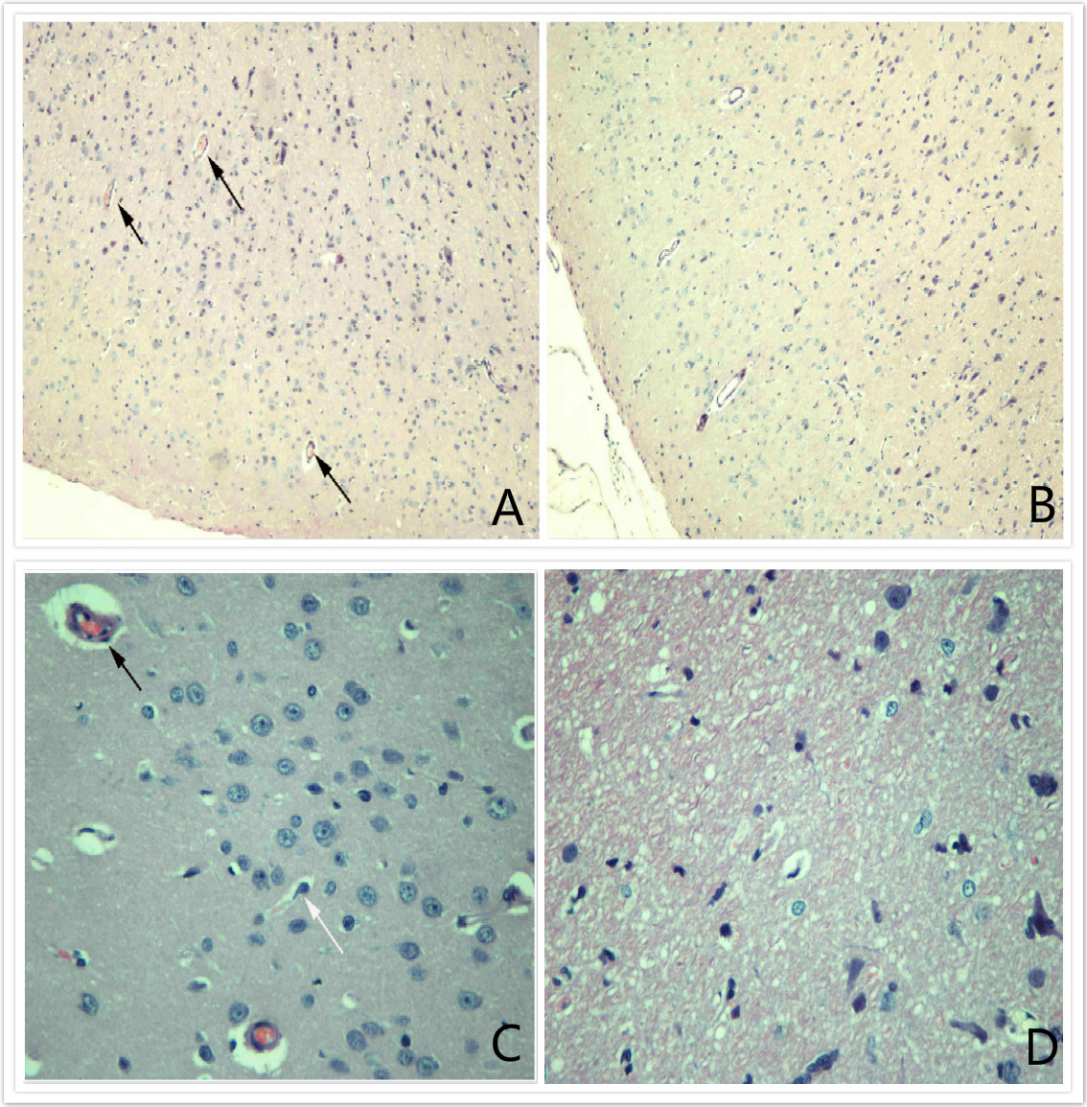
HE (X100) staining **A.** and **C.** reveal the presence of certain amounts of thrombi in microvessels and large numbers of pyknotic neurons in the ipsilateral cerebral tissue; **B.** and **D.** show that no thrombi were present in microvessels in the lateral cerebral tissue after compression injury.

After hematoxylin and eosin (H&E) staining, optical microscopic observation revealed the presence of a certain amount of thrombi in microvessels and large numbers of pyknotic neurons in the ipsilateral cerebral tissue but not in the lateral cerebral tissue after compression injury (Figure 6).

## DISCUSSION

An increasing amount of attention has been paid to post-traumatic hemodynamic monitoring in modern neurosurgery. However, the research and interventions on secondary brain injury after TBI have mainly relied on the monitoring of parameters such as ICP, CPP, and CBF. In recent years, various studies have confirmed that for both TBI animal models and patients with TBI, simultaneous monitoring of multiple parameters, including ICP, CPP, and CBF, provides more values than the monitoring of a single parameter [6].

CEU is a newly developed technology that uses a microbubble contrast agent to significantly enhance ultrasound signals. The intravenously injected contrast agent can reach every organ in the body and can significantly improve the sensitivity of detection for even fine trickles of blood. This technology reduces the background noise and increases the signal-to-noise ratio and can be used to evaluate blood perfusion through analysis of the signal intensity-to-time curve. Compared with other monitoring technologies, CEU is easier to operate and can provide a real-time, non-invasive, quantitative, and intuitive presentation of blood perfusion in the regional microcirculation. This technology has been widely used in various systems [7], and its effectiveness in evaluating rCBF has been validated by domestic and international researchers [8, 9]. The experimental studies during the same period also confirmed that CEU could quantitatively evaluate rCBF (A×β) [9] through measurements of the microcirculatory blood flow volume (A) and velocity (β). Therefore, using an epidural brain compression injury model that closely mimics actual clinical scenarios, the present study adopted a ‘gold standard’ ventricular ICP monitoring technology to detect ICP and CEU to monitor rCBF [10]. Our findings can provide a foundation for further exploration of the relationship between CPP and rCBF.

Kasapas et al [11] built a successful epidural hematoma model on rabbits, from their research, the conclusion was that invasive ICP increments in an experimental model of epidural hematoma resulted in doppler derived pulsatility index, optic nerve sheath diameter measurements, and so on. We built a successful epidural hematoma model on dogs[12]. In the hyper-acute phase (within 2 hours), ICP increased significantly when the volume of sacculus was around 1.5 ml (20 min after injection), CPP gradually reduced according to the increase of the volume of sacculus. When the volume of sacculus was around 7ml, MAP increased dramatically, howerver, there was no difference such as PaO2, PaCO2, PH and SaO2.

Our data showed that the rCBF value might be influenced by the rCPP, while the latter varied at different locations; thus, the rCBF merely reflects the rCPP level. The relationship between the rCPP and global CPP needs to be further investigated. We believe that ICP and CPP monitoring alone cannot truly reflect the change in rCBF. To date, there has been no definitive conclusion regarding the existence of a specific relationship between the CPP and rCBF; instead, the latter is believed to have a possibly closer relationship with the rCPP. The question about whether the rCBF has a linear relationship or other type of relationship with the CPP remains to be clarified. However, it is certain that an accurate assessment of the self-regulatory function of cerebral vessels can reveal the true relationship between the rCBF and CPP because the rCBF is regulated through the control of microvascular contraction and relaxation via a self-regulatory mechanism.

For both diffuse and focal brain injuries, decreased rCBF at the early stage after injury has been confirmed. In 1965, Lundberd [13] reported that CBF might vary over time and at different locations after TBI. Through vascular Doppler imaging in rats, Plesnila [14] found that within 6 h after brain contusion and laceration, the CBF in the injured area decreased to 30% of the initial level, while the CBF in the surrounding non-injured area decreased to 80% of the initial level, confirming the spatial variation in the post-traumatic CBF. Therefore, the concept of ischemic penumbra that was originally suggested by the research on cerebral stroke was introduced to the TBI research on cerebral trauma surgery and was called “traumatic penumbra”. The penumbra zone surrounding focal traumatic brain lesions is fully reversible. Therefore, attaining an understanding of the penumbra can effectively guide the treatment to rescue the cerebral tissue in this zone. In the present study, we observed a decrease in the rCBF in the area surrounding the compression site since the beginning of compression. Moreover, the degree of the rCBF decrease varied significantly among different locations. The rCBF at sites 1 cm away from the compression site decreased remarkably more than that at sites 2 cm away from the compression site, as evidenced by the fact that the former reached zero, while the latter decreased by less than 40%. This significant difference under the same CPP indicated a spatial difference in the regional blood supply in the surrounding area of traumatic brain lesions, i.e., the rCBF in the area farther from the lesions was subject to less of a compression effect. This finding is another piece of evidence supporting the aforementioned conclusion. A possible mechanism might be that as ICP increases, the cerebral vascular regulation is initiated earlier and affects the regional vascular self-regulatory function more severely in the area closer to the lesion, resulting in a gradient in the rCPP reduction and a subsequent gradient in the decrease in rCBF. From a clinical perspective, Schmidt [15] confirmed the cerebral hemispheric asymmetry of the vascular self-regulatory function and stated that the self-regulatory function deteriorates more severely and leads to worse outcomes in the area farther from the injured cerebral region. This finding indirectly supports our conclusion.

Cerebral microthrombi are extensively distributed on both the ipsilateral and collateral sides of the brain after TBI. These microthrombi are mostly concentrated in the traumatic lesions and their marginal areas on the ipsilateral side. On the collateral side, thrombi can be extensive but at a much lower density compared with those on the ipsilateral side. In addition, the amounts of microthrombi are usually greater after focal and severe brain injuries but are relatively lower after diffuse and minor brain injuries [16] An experimental study on rats showed that thrombosis appeared at 1-4 h post-TBI, peaked at 1-3 days post-TBI, and began to decrease at 8-15 days post-TBI. Cerebral microthrombosis is common in TBI patients, but very few studies have investigated its epidemiological distribution pattern due to the complexity of the influencing factors.

Our pathological sectioning revealed a large amount of microthrombi accompanied by cell apoptosis in the microvessels in the ipsilateral cerebral cortex, while a similar phenomenon was not observed in the collateral cerebral cortex after injury. This finding is consistent with previous reports. For example, Huber [17] observed brain specimens of TBI patients and found a larger amount of microthrombi on the ipsilateral side than on the collateral side. Similar findings were also reported by Stein [18], who investigated the pathological brain specimens of patients who died of TBI.

We believe that post-TBI microthrombosis can be attributed to the endothelial cell damage associated with the microvascular displacement due to the increased ICP. In addition, Liu [19] analyzed the factors related to the post-TBI hemorheological changes and found that patients with higher ICP exhibited more prominent hemorheological changes, mainly hyperviscosity without coagulation anomalies. The causal relationship between high ICP and hyperviscosity might involve the increased activity of the sympathetic-adrenal system caused by high ICP, which leads to extensive contraction of peripheral small arteries and those supplying the spleen and ultimately results in hemoconcentration and hyperviscosity. This effect might be one of the factors promoting post-traumatic microthrombosis.

## MATERIALS AND METHODS

### Animals and acute epidural hematoma model

All procedures that involved animals were conducted under the National Guidelines for the Care and Use of Laboratory Animals and the Animal Care Guidelines issued by the Animal Experimental Committee of Beijing Neurosurgical Institute. Experiments were performed on 10 mongrel dogs (weight 18.3 ± 1.6 kg) that were fasted overnight with free access to water. Anesthesia was provided with 3% nembutal. After orotracheal intubation, mechanical ventilation was initiated. The femoral artery and vein were cannulated, and pulmonary artery 7F catheters were inserted separately and connected to a cardiac output monitor. The left parietal lobe was opened with 4.0 cm * 2.5 cm incision to display the dura. An ICP sensor was placed in the right parietal lobe through a 5-mm burr hole drilled in the parietal bone and was connected to a ICP. An 8-mm diameter hole was also drilled into the right parietal bone for the insertion of an epidural latex sacculus. Saline was injected into the epidural latex sacculus at a rate of 4.5 ml/h. We used the formulation (CPP = MAP [mean arterial pressure] - ICP) to calculate CPP until the CPP approached zero. The animal’s core temperature was continuously monitored and maintained at 36-38°C by a negative-feedback-controlled heating pad during the entire experiment.

### Contrast-enhanced ultrasound

Using a Logiq9 ultrasound by Pulse Inversion Harmonics (PIH), with a probe frequency of 2.5-4.5 MHz, the area around the balloon, 1 and 2 cm within the left bone window, was selected as the region of interest (ROI). After adjusting the image, the position and angle of the ultrasound probe and the depth selected remained unchanged. Microbubbles (Sonovue, Bracco, Italy) were dissolved in 6 ml of saline (4.17 mg/ml) and mixed. The ICP increased for every 10 mmHg; a bolus injection of 2 ml contrast agent and 5 ml saline was administered; simultaneously, the timer was started in the ultrasound system, and real-time perfusion in the ROI was observed. Continuous observations were made over 4 mins, and images were stored.

### Image analysis

Image analysis software for contrast echocardiography was used to analyze the images. According to the exponential equation Y = A × (1 - e^-βt^), the relationship between the video intensity of the ROI and the perfusion time of the contrast agent was plotted. CEU parameters, including the A, β, and A×β values, were automatically provided; A is the maximum number of contrast agent microbubbles that can accumulate in the regional tissue, β is the filling speed of contrast agent microbubbles in the regional tissue, and A×β refers to the volume of the regional blood flow.

### Staining

The animals subjected to trauma were killed 2 h after trauma by transcardial perfusion with 4% paraformaldehyde solution under deep halothane anesthesia. Brains were postfixed in 4% paraformaldehyde overnight, dehydrated, and embedded in paraffin. Brain sections were stained with hematoxylin and eosin to examine morphology. Briefly, after washing the brain sections in PBS (0.01 mol/L, pH 7.3), they were stained in hematoxylin solution (Sigma-Aldrich, MO, USA) for 5 mins and washed under running tap water for 1 min. Sections were then differentiated in 1% acid alcohol and stained blue in 0.2% ammonia water solution, followed by counterstaining with 0.5% eosin (Sigma-Aldrich, MO, USA) for 5 minutes, washed off, dehydrated in progressive alcohol concentrations, cleared in xylene, and mounted with Permount (Fisher Scientific, NJ, USA).

### Statistical analysis

All data are expressed as the means ± standard deviations and were statistically analyzed using GraphPad Prism (version 6.04, GraphPad Siftware Inc). To elucidate the relationship between CPP and rCBF, curvilinear regression/correlation analyses were conducted to determine the correlation coefficient and regression equation. Paired t tests were performed to investigate the differences between the CBF values at different locations under the same CPP.

## References

[1] Clifton GL, Miller ER, Choi SC, Levin HS, McCauley S, Smith KR, Muizelaar JP, Wagner FC, Marion DW, Luerssen TG, Chesnut RM, Schwartz M (2001). Lack of effect of induction of hypothermia after acute brain injury. N Engl J Med.

[2] Graham DI, Adams JH, Doyle D (1978). Ischaemic brain damage in fatal non-missile head injuries. J Neurol Sci.

[3] Hakim AM (1998). Ischemic penumbra: the therapeutic window. Neurology.

[4] Rosner MJ, Rosner SD, Johnson AH (1995). Cerebral perfusion pressure: management protocol and clinical results. J Neurosurg.

[5] Muizelaar JP, Lutz HA, Becker DP (1984). Effect of mannitol on ICP and CBF and correlation with pressure autoregulation in severely head-injured patients. J Neurosurg.

[6] Juul N, Morris GF, Marshall SB, Marshall LF, The Executive Committee of the International Selfotel Trial (2000). Intracranial hypertension and cerebral perfusion pressure: influence on neurological deterioration and outcome in severe head injury. J Neurosurg.

[7] Xie JG, Liu YL, Zha DG, Bin JP, Liu J, Wu PS (2005). [An experimental study on renal microvascular perfusion in dogs with acute cardiac insufficiency]. [Article in Chinese]. Zhonghua Xin Xue Guan Bing Za Zhi.

[8] Rim SJ, Leong-Poi H, Lindner JR, Couture D, Ellegala D, Mason H, Durieux M, Kassel NF, Kaul S (2001). Quantification of cerebral perfusion with “Real-Time” contrast-enhanced ultrasound. Circulation.

[9] Zeng P, Zha D, Bin J, Zhou Y, Chen Y, Liu Y (2010). Effects of acute cerebral ischemia on cerebral perfusion: quantitative evaluation by contrast-enhanced ultrasound in dogs. J South Med Univ.

[10] Procaccio F, Stocchetti N, Citerio G, Berardino M, Beretta L, Della Corte F, D’Avella D, Brambilla GL, Delfini R, Servadei F, Tomei G (2000). Guidelines for the treatment of adults with severe head trauma (part I). Initial assessment; evaluation and pre-hospital treatment; current criteria for hospital admission; systemic and cerebral monitoring. J Neurosurg Sci.

[11] Kasapas K, Diamantopoulou A, Pentilas N, Papalois A, Douzinas E, Kouraklis G, Slama M, Terkawi AS, Blaivas M, Sargsyan AE, Karakitsos D (2014). Invasive and ultrasound based monitoring of the intracranial pressure in an experimental model of epidural hematoma progressing towards brain tamponade on rabbits. Sci World J.

[12] Xu F, Liu H, Shi G, Liu B (2011). Establishment of refractory high intracranial pressure model in dogs. Chinese Journal of Neurosurgery.

[13] Lundberg N, Troupp H, Lorin H (1965). Continuous recording of the ventricular-fluid pressure in patients with severe acute traumatic brain injury. A preliminary report. J Neurosurg.

[14] Plesnila N, Friedrich D, Eriskat J, Baethmann A, Stoffel M (2003). Relative cerebral blood flow during the secondary expansion of a cortical lesion in rats. Neurosci Lett.

[15] Schmidt EA, Czosnyka M, Steiner LA, Balestreri M, Smielewski P, Piechnik SK, Matta BF, Pickard JD (2003). Asymmetry of pressure autoregulation after traumatic brain injury. J Neurosurg.

[16] Lu D, Mahmood A, Goussev A, Qu C, Zhang ZG, Chopp M (2004). Delayed thrombosis after traumatic brain injury in rats. J Neurotrauma.

[17] Huber A, Dorn A, Witzmann A, Cervos-Navarro J (1993). Microthrombi formation after severe head trauma. Int J Legal Med.

[18] Stein SC, Graham DI, Chen XH, Smith DH (2004). Association between intravascular microthrombosis and cerebral ischemia in traumatic brain injury. Neurosurgery.

[19] Liu B, Fu Z, Yu C, Huang Y, Bao T (2000). Analysis of the relative factors affecting hemorheological changes following head injury Chin J. Crit Care Med.

